# Effect of Red Light Rhinophototherapy on Nasal Patency in Patients with Allergic Rhinitis

**DOI:** 10.1155/2018/6270614

**Published:** 2018-12-17

**Authors:** Rong-San Jiang, Jing-Jie Wang

**Affiliations:** ^1^Department of Medical Research, Taichung Veterans General Hospital, Taichung, Taiwan; ^2^Department of Otolaryngology, Taichung Veterans General Hospital, Taichung, Taiwan; ^3^School of Medicine, Chung Shan Medical University, Taichung, Taiwan; ^4^Rong Hsing Research Center For Translational Medicine, National Chung Hsing University, Taichung, Taiwan; ^5^Institute of Medicine, Chung Shan Medical University, Taichung, Taiwan

## Abstract

**Purpose:**

The effect of red light rhinophototherapy (RLRPT) on nasal patency in patients with a clinical diagnosis of allergic rhinitis was investigated.

**Materials and Methods:**

Subjects were randomly divided into 2 groups, with patients in one group given one treatment session of RLRPT, followed by medical treatment. Those in the second group were treated with medical treatment only. The rhinitis symptoms were evaluated both before and 30 minutes after RLRPT and 2 days later. The nasal patency was objectively measured through the use of both active anterior rhinomanometry and acoustic rhinometry before and 30 minutes after RLRPT.

**Results:**

All rhinitis symptoms, including nasal congestion, significantly improved 30 minutes after a single RLRPT treatment, but worsened again, particularly for sneezing, 2 days later. Nasal resistance slightly decreased 30 minutes after RLRPT. The first minimal cross-sectional area did not change after RLRPT, but the second minimal cross-sectional area with the volume of the nasal cavity between 2.0 and 5.0 cm from the tip of the nosepiece significantly lessened.

**Conclusions:**

This study showed that RLRPL did not objectively improve patient's nasal patency.

**Registration Number:**

The trial is registered with NCT03752645.

## 1. Introduction

Rhinophototherapy has been used to treat both allergic rhinitis (AR) and chronic rhinosinusitis [1, 2]. Three different devices for rhinophototherapy have been developed [3]. One device emits a visible red light at a single wavelength of 660 nm (red light rhinophototherapy, RLRPT). The second device emits an infrared light at wavelengths of 652 nm and 940nm. The third device emits a composite light which consists of 70% visible light, 25% ultraviolet light-A, and 5% ultraviolet light-B.

It has been suggested that rhinophototherapy could relieve the nasal symptoms of AR [4]. One study has shown that after the nasal cavity was illuminated using a low-energy narrow-band red light three times a day for 14 days, the symptoms and endoscopic findings in patients with allergic rhinitis improved significantly [1]. When the mechanism of rhinophototherapy is not fully understood, a low-energy narrow-band light illumination has been claimed to have biochemical, cellular, histological, and functional effects [1]. The heat generated by light illumination may alter the mucosal blood circulation in the nasal cavity [5]. It has been reported that rhinophototherapy has reduced nasal obstruction more successfully than an antihistamine nasal spray [6]. Another report has also shown that rhinophototherapy could increase nasal inspiratory peak flow [7]. However, the effect of rhinophototherapy on the nasal patency has not yet been fully investigated. In this study, we attempted to investigate the short-term effects of RLRPT on nasal patency in patients with a clinical diagnosis of AR using both active anterior rhinomanometry and acoustic rhinometry.

## 2. Materials and Methods

This study was approved by the Ethics Committee of Taichung Veterans General Hospital. Written consent was obtained from each patient.

### 2.1. Study Population

Patients experiencing moderate to severe symptoms of allergic rhinitis were collected from the outpatient clinic of the Department of Otolaryngology between March of 2018 and June of 2018. The clinical diagnosis of allergic rhinitis was based on the Clinical Practice Guideline: Allergic Rhinitis publication from the American Academy of Otolaryngology Head-Neck Surgery Foundation [6]. The clinical diagnosis of AR was established when patients presented themselves with a history and physical examination consistent with an allergic cause. Each candidate possessed at least one of the following symptoms: nasal congestion, runny nose, itchy nose, or sneezing. Additionally, a physical examination found clear rhinorrhea, nasal congestion, pale discoloration of the nasal mucosa, or red and watery eyes in each patient.

All patients underwent a specific IgE test against the common perennial inhaled allergens found in Taiwan (house dust mites, molds, cats, dogs, and cockroaches) to confirm the diagnosis of AR. However, these results did not exclude the patients from this study because only a few allergens were tested. The severity of the rhinitis symptoms was assessed through use of a standardized score scale (1). A score of 0 (no symptoms), 1 (mild symptoms), 2 (moderate symptoms), to 3 (severe symptoms) was used to evaluate the severity of nasal congestion, runny nose, itchy nose, and sneezing. Patients receiving a total score of 4 or more were enrolled in the study. Patients with age below 20 years, severe deviated nasal septum, rhinosinusitis, and nasal polyposis were excluded from the study. Those who had a history of immunodeficiency or previous sinus surgery, suffered from an upper respiratory tract infection, or took oral corticosteroids within a month prior to the study were also excluded.

### 2.2. Study Design

Eligible patients were randomly divided into 2 groups. Randomization assignments were generated by an independent statistician. Patients in the study group were treated with one treatment session of RLRPT (40mW/nostril for 15 minutes) at the outpatient clinic after completing a nasal patency test using both active anterior rhinomanometry and acoustic rhinometry. Upon completing RLRPT treatment, patients took a rest for 30 minutes. They were then asked about the severity of their rhinitis symptoms, and as to whether the overall level of change in those rhinitis symptoms was worse, unchanged, slightly improved, much improved, or cured. Patients were also questioned about any adverse events of RLRPT before undergoing another nasal patency test. Finally, medical treatment involving an intranasal steroid (mometasone furoate nasal spray, 4 sprays, once a day), along with an oral antihistamine (levocetirizine 5 mg qd) was given for continued management of AR. Questions regarding the severity of each patient's rhinitis symptoms, the overall change in their rhinitis symptoms, and any adverse events from RLRPT were asked via telephone communication 2 days later. Patients in the active control group were medically treated with an intranasal steroid (mometasone furoate nasal spray, 4 sprays, once a day), along with an oral antihistamine (levocetirizine 5 mg qd). Telephone calls were placed 2 days later in order to evaluate the severity of each patient's rhinitis symptoms, along with any overall change in rhinitis symptoms.

### 2.3. Red Light Rhinophototherapy

**Figure 1:**
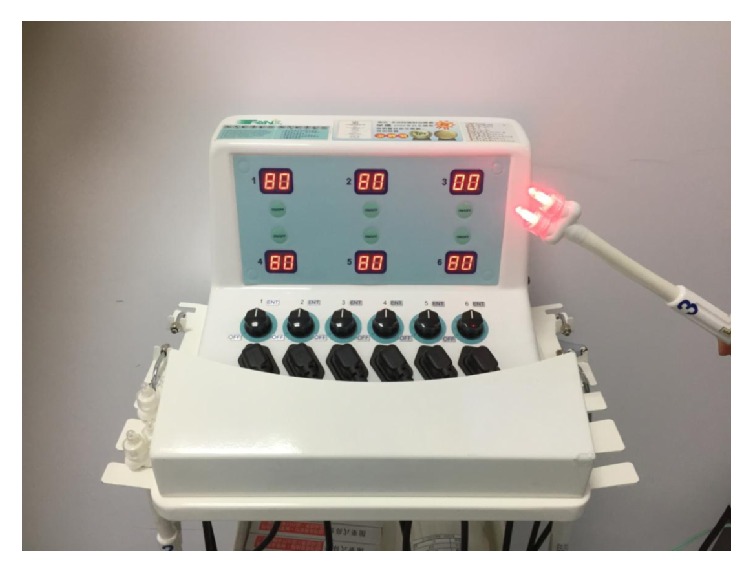
Transverse Many Channels Laser Instrument (Transverse, Ind, Co., Ltd., Taipei, Taiwan).

**Figure 2:**
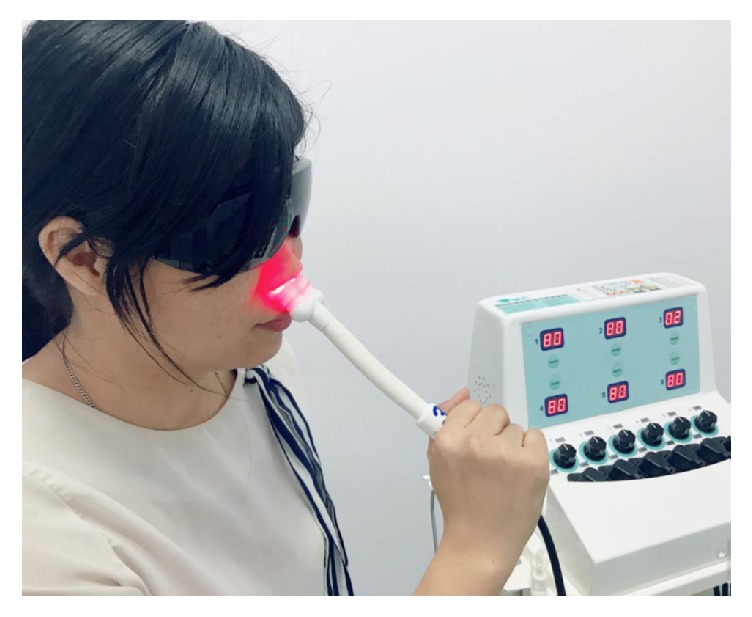
A patient receiving red light rhinophototherapy through two light-emitting nasal probes.

The device used for RLRPT was the Transverse Many Channels Laser Instrument (Transverse, Ind, Co., Ltd., Taipei, Taiwan) (Figure 1). It uses a red gallium-aluminum-arsenide laser with wavelengths of 660±10 nm as a light source [7]. The laser has a maximum power of 40 mW. The device consists of a control box and 4 sets of two light-emitting nasal probes. Prior to treatment, each patient put on a pair of black tinted glasses and had the nasal probes gently placed into both nostrils (Figure 2). A turn-on switch on the control box activated the probe and the timer was set at 15 minutes during which time 36 J of light energy was delivered to each nostril.

### 2.4. Nasal Patency Test

The nasal patency was objectively measured by both active anterior rhinomanometry and acoustic rhinometry. Anterior active rhinomanometry was performed according to the guidelines of the International Committee on Standardization of Rhinomanometry using a NR6 Rhinomanometer (GM Instruments, Ltd., Kilwinning, UK) [8]. All patients remained seated for 30 minutes to adapt to the hospital environment prior to testing. A face mask was worn tightly. The examination was performed during quiet breathing with a closed mouth, while the patient was in an upright sitting position. For each nostril, inspiratory nasal resistance was calculated over four inspiratory-expiratory cycles at a fixed pressure of 75 Pascal. Both the total nasal airflow resistance in Pa/cm3/s and nasal airflow in cm^3^/s (sum of left and right) during inspiration were recorded.

An A1 Acoustic Rhinometer (GM Instruments, Ltd., Kilwinning, UK) was used to measure the geometry of the nasal cavity [9, 10]. All patients remained seated for at least 20 minutes in order to acclimatize to the hospital environment before testing. The nose piece was positioned parallel to the sagittal plane of the head at a 45° angle to the coronal plane and was applied to produce an acoustic seal without distorting the outer nose. Patients were asked to hold their breath and avoid swallowing during the acquisition of the acoustic data. Three consecutive readings were taken to calculate an average value. An entire average acoustic rhinometry curve was generated for each nasal cavity. Acoustic data included (1) the first minimal cross-sectional area (MCA_1_, cm^2^), (2) the second minimal cross-sectional area (MCA_2_, cm^2^), (3) the volume between the tip of the nosepiece and 3.0 cm into the nasal cavity (V03, cm^3^), and (4) the volume of the nasal cavity between 2.0 and 5.0 cm from the tip of the nosepiece (V25, cm^3^). We used the average value of both sides to represent the data.

### 2.5. Statistical Analysis

All data is presented as mean ± standard deviation. Patient's gender was compared between the two groups using the Chi-square test. The ages and rhinitis symptom scores during the visit and 2 days after the visit were compared between the two groups using the Mann-Whiney U test. The rhinitis symptoms in the study group were compared before RLRPT, 30 minutes after RLRPT, and 2 days after RLRPT using the Wilcoxon signed-rank test. Rhinitis symptoms in the active control group were compared during the visit and 2 days after the visit using the Wilcoxon signed-rank test. Total nasal resistance, total nasal flow, MCA1, MCA_2_, V03, and V25 were compared between before RLRPT and 30 minutes after RLRPT using the Wilcoxon signed-rank test. It was considered statistically significant when p-values were < 0.05. A SPSS version 17.0 (SPSS Inc., Chicago, IL, USA) was used to perform all analyses.

## 3. Results

### 3.1. Demographic Data

Sixty patients with a clinical diagnosis of allergic rhinitis were enrolled between March and June of 2018. There were 18 males and 12 females in the study group, with 20 males and 10 females in the active control group. The mean age was 45.4 years with a range of 20 to 87 years in the study group and a mean age of 45.7 years with a range of 20 to 88 years in the active control group. There were no significant differences in gender and age (p=0.789, 0.712, respectively). Seventeen patients in the study group and 18 in the active control group experienced a positive specific IgE test against the common perennial inhaled allergens found in Taiwan.

### 3.2. Comparison of Symptom Scores before and after Treatment

All rhinitis symptoms significantly improved 30 minutes after RLRPT (Table 1). One patient felt his symptoms had been cured after RLRPT, 6 reported their symptoms were much improved, 12 claimed their symptoms had slightly improved, with the remaining 11 claiming that their symptoms were unchanged. Two days following medical treatment, all rhinitis symptoms remained significantly better than prior to treatment (Table 2). Seven patients reported that their symptoms were much improved, 16 claimed their symptoms had slightly improved, and the symptoms in the remaining 7 remained unchanged. When comparing scores between 30 minutes after RLRPT and 2 days after RLRPT, rhinitis symptoms became worse overall 2 days after RLRPT, particularly in regard to sneezing (p=0.011) (Tables 1 and 2).

All rhinitis symptoms also significantly improved 2 days after medical treatment in the active control group (Table 3). Eleven patients reported that their symptoms were much improved, 10 said their symptoms had slightly improved, with the symptoms being unchanged in the remaining nine. After the rhinitis symptom scores were compared between two groups prior to treatment and 2 days after treatment, no significant differences were found in any of the rhinitis symptoms.

### 3.3. Comparison of Nasal Patency before and after Treatment

Nasal resistance slightly decreased, while nasal airflow slightly increased 30 minutes after RLRPT, when compared with prior to RLRPT (Table 1). The MCA_1_ did not change after RLRPT, but MCA_2_ with V25 significantly lessened (Table 1).

### 3.4. Adverse Events

RLRPT with wavelengths of 660 nm at a power of 40 mW for a 15-minute illumination period was well tolerated by most patients. One patient felt a burning sensation around the nostril 30 minutes after RLRPT and still felt pain around the nostril 2 days later. Another had pain around the nostril 30 minutes after RLRPT but the pain went away 2 days later. Another two suffered from mild headaches 30 minutes after RLRPT, but their headaches soon disappeared.

## 4. Discussion

Rhinophototherapy has been recommended into the ARIA guidelines for those patients with allergic rhinitis who do not respond to standard medical treatment [11]. It is considered to have a strong immunosuppressive effect [12]. The main mechanisms involve apoptosis of T lymphocytes and eosinophils, reduction in the number and function of dendritic cells, and induction of immunomodulatory cytokines such as IL-10 [13]. RLRPT has been claimed to generate some heat apart from the light. The heat may modulate mucosal blood supply and histamine release [5].

Tatar et al. reported that rhinophototherapy paired with medical therapy (topical mometasone furoate and oral levocetirizine) had a better effect on allergic rhinitis symptoms, including nasal obstruction, than did medical therapy on its own [14]. Our results show that all rhinitis symptoms, including nasal congestion, significantly improved 30 minutes after a single RLRPT treatment, but worsened again, particularly for sneezing, 2 days later even under medical treatment. This indicates that RLRPT was effective in improving rhinitis symptoms, but the effect was not long lasting. Usually, treatment involved the nasal cavities being illuminated from twice daily to twice a week, for at least 2 weeks [5, 6, 12].

It has been reported that nasal obstruction was improved by rhinophototherapy; however objective evaluation was not included in most studies. Albu and Baschir [6] used active anterior rhinomanometry to measure nasal resistance before and after rhinophototherapy. The nasal resistance in their subjects decreased after rhinophototherapy as our results, but the decrease was not significant in either study.

When acoustic rhinometry is used to measure the geometry of the nasal cavity, MCA_1_ is considered to correspond to the nasal valve, and is 0.5 to 1.0 cm from the nasal inlet. MCA_2_ corresponds to the anterior half of the inferior turbinate which contains much cavernous erectile tissue, and is 2.0 to 5.0 cm from the tip of the nosepiece [15]. Therefore, MCA_2_, V03 and V25, but not MCA_1_ often increased after nasal decongestion [9]. In contrast, our results show that MCA_2_ and V25 decreased after RLRPT.

RLRPT has been claimed to generate some heat which alters mucosal blood supply. The size of the edematous congestion of the inferior turbinate and the amount of nasal discharge were observed to become decreased after RLRPT, through use of a nasal endoscopy [5, 12]. Our results of acoustic rhinometry indicate that one-time RLRPT treatment did not immediately open up the nasal cavity. The improvement in the symptom of nasal congestion and a slight decrease in nasal resistance should come from different mechanisms. For example, a decrease in the amount of nasal discharge after RLRPT may help in decreasing both nasal congestion and nasal resistance.

Dry nostrils have been reported to be the most common adverse event of rhinophototherapy [6, 7]. Our patients did not complain of nasal dryness partly because they only received one RLRPT treatment. Headaches and nasal burning sensation were other reported side effects which also occurred in a few of our patients [13]. Other possible adverse events such as septal perforation and epistaxis did not occur in our patients [12]. Overall, RLRPT was well tolerated by our patients.

There were some limitations in our study. The improvement of rhinitis symptoms after RLRPT may have been affected by placebo-effects [6]. When patients in the study group received a more active intervention of RLRPT, they were prone to favor RLRPT. In this study, we evaluated the effect of one RLRPT treatment on nasal patency. The standard treatment protocol usually lasts from twice daily to twice a week for at least 2 weeks [5, 6, 12]. Therefore, the actual effect of RLRPT on nasal patency still requires further investigation. Finally, we only included adult patients. The effect of RLRPT on children with allergic rhinitis needs further study too.

## 5. Conclusions

In this study, we evaluated the effect of one RLRPT treatment on rhinitis symptoms and nasal patency. Our results show that all rhinitis symptoms, including nasal congestion, significantly improved after a single RLRPT treatment. However, the improvement of rhinitis symptoms after RLRPT may have been affected by placebo-effects. On the other hand, one RLRPT treatment did not objectively improve patient's nasal patency, but the actual effect of RLRPT on nasal patency still requires further investigation.

## Figures and Tables

**Table 1:** Comparison before and 30 minutes after red light rhinophototherapy (RLRPT).

Symptoms/Nasal patency	Before RLRPT	30 minutes after RLRPT	P-value
Nasal congestion	1.83±0.91	1.13±1.01	<0.0001
Runny nose	1.57±0.73	0.47±0.82	<0.0001
Itchy nose	1.20±0.89	0.50±0.73	<0.0001
Sneezing	1.40±0.72	0.13±0.43	<0.0001
Total rhinitis score	6.00±1.53	2.23±1.59	<0.0001
Nasal resistance	0.32±0.23	0.28±0.11	0.245
Nasal flow	293.71±133.22	317.47±121.25	0.156
MCA_1_	0.71±0.11	0.71±0.06	0.674
MCA_2_	0.44±0.21	0.36±0.17	0.006
V03	2.22±0.51	2.13±0.40	0.09
V25	3.38±1.45	2.79±0.94	0.001

MCA_1_: the first minimal cross sectional area. MCA_2_: the second minimal cross sectional area. V03: the volume between the tip of the nosepiece and 3.0 cm into the nasal cavity. V25: the volume of the nasal cavity between 2.0 and 5.0 cm from the tip of the nosepiece.

**Table 2:** Comparison before and 2 days after red light rhinophototherapy (RLRPT).

Symptoms/Nasal patency	Before RLRPT	2 days after RLRPT	P-value
Nasal congestion	1.83±0.91	1.17±0.91	0.001
Runny nose	1.57±0.73	0.87±0.94	0.004
Itchy nose	1.20±0.89	0.50±0.73	0.001
Sneezing	1.40±0.72	0.53±0.78	<0.0001
Total rhinitis score	6.00±1.53	3.07±2.1	<0.0001

**Table 3:** Comparison before and 2 days after medical treatment in the control group.

Symptoms/Nasal patency	Before RLRPT	2 days after RLRPT	P-value
Nasal congestion	2.07±0.98	1.40±1.00	<0.0001
Runny nose	1.73±0.91	0.87±0.86	<0.0001
Itchy nose	1.30±1.12	0.77±0.97	0.003
Sneezing	1.63±0.93	0.70±0.84	<0.0001
Total rhinitis score	6.73±2.32	3.73±2.63	<0.0001

## Data Availability

The data used to support the findings of this study are available from the first author upon request.
